# Interrogating and Reflecting on Disability Prevalence Data Collected Using the Washington Group Tools: Results from Population-Based Surveys in Cameroon, Guatemala, India, Maldives, Nepal, Turkey and Vanuatu

**DOI:** 10.3390/ijerph18179213

**Published:** 2021-08-31

**Authors:** Islay Mactaggart, Ammar Hasan Bek, Lena Morgon Banks, Tess Bright, Carlos Dionicio, Shaffa Hameed, Shailes Neupane, GVS Murthy, Ahmed Orucu, Joseph Oye, Jonathan Naber, Tom Shakespeare, Andrea Patterson, Sarah Polack, Hannah Kuper

**Affiliations:** 1 International Centre for Evidence in Disability, London School of Hygiene & Tropical Medicine, Keppel Street, London WC1E 7HT, UK; morgon.banks@lshtm.ac.uk (L.M.B.); Tess.Bright@lshtm.ac.uk (T.B.); Shaffa.Hameed@lshtm.ac.uk (S.H.); tom.shakespeare@lshtm.ac.uk (T.S.); Sarah.Polack@lshtm.ac.uk (S.P.); Hannah.Kuper@lshtm.ac.uk (H.K.); 2 Relief International, Istanbul 34087, Turkey; ammar.hasanbek@ri.org (A.H.B.); andrea.patterson@ri.org (A.P.); 3 Center for Research in Indigenous Health, Wuqu’ Kawoq, Maya Health Alliance 2a Avenida 3-48 Zona 3, Barrio Patacabaj, Tecpán, Chimaltenango 4001, Guatemala; dr.carlosdionicio@gmail.com; 4 Valley Research Group, Kathmandu 44600, Nepal; shailes@varg.wlink.com.np; 5 Indian Institute of Public Health, Hyderabad 122002, India; gvs.murthy@lshtm.ac.uk; 6 Mülteciler Derneği, Istanbul 34930, Turkey; ahmed.orucu@multeciler.org.tr; 7 Sightsavers Cameroon, Yaounde P.O. Box 4484, Cameroon; joye@sightsavers.org; 8 Range of Motion Project, P.O. Box 100915, Denver, CO 80250, USA; jonathan@rompglobal.org

**Keywords:** disability measurement, population surveys, disability prevalence

## Abstract

The Washington Group (WG) tools capture self-reported functional limitations, ranging from 6 domains in the Short Set (SS) to 11 in the Extended Set (ESF). Prevalence estimates can vary considerably on account of differences between modules and the different applications of them. We compare prevalence estimates by WG module, threshold, application and domain to explore these nuances and consider whether alternative combinations of questions may be valuable in reduced sets. We conducted secondary analyses of seven population-based surveys (analyses restricted to adults 18+) in Low- and Middle-Income Countries that used the WG tools. The prevalence estimates using the SS standard threshold (a lot of difficulty or higher in one or more domain) varied between 3.2% (95% Confidence Interval 2.9–3.6) in Vanuatu to 14.1% (12.2–16.2) in Turkey. The prevalence was higher using the ESF than the SS, and much higher (5 to 10-fold) using a wider threshold of “some” or greater difficulty. Two of the SS domains (communication, self-care) identified few additional individuals with functional limitations. An alternative SS replacing these domains with the psychosocial domains of anxiety and depression would identify more participants with functional limitations for the same number of items. The WG tools are valuable for collecting harmonised population data on disability. It is important that the impact on prevalence of use of different modules, thresholds and applications is recognised. An alternative SS may capture a greater proportion of people with functional domains without increasing the number of items.

## 1. Introduction

Disability is a complex phenomenon that has been historically difficult to define. It is an umbrella term for the functional limitations that result from a health condition (e.g., glaucoma) interacting with contextual factors (e.g., access to assistive products or enabling environments) [1,2]. Functional limitations can be experienced at the level of the body (impairments, e.g., disorders of the eye), person (activity limitations, e.g., seeing) or society (participation restrictions, e.g., going to work).

The global prevalence of disability is commonly cited as 15%, as proposed in the 2011 World Report on Disability [3,4,5]. This estimate is based on pooled analyses of the 2002–2004 World Health Surveys, which included self-reported data on functional limitations in 8 “life domains” (including mobility, sleep and energy, and interpersonal activities) from adults aged 18 and above across 59 countries [4]. This figure has been central to global advocacy efforts since its publication and has been leveraged by disability stakeholders to encourage policy makers, development donors, the private sector and the general public to dedicate appropriate resources and attention to disability inclusion [6,7]. However, the methodology used to arrive at the estimate is not directly comparable to prevalence estimates derived from population-based surveys of disability using prevailing methods.

The dominant prevailing methodology for estimating disability prevalence in a given population is to use the Washington Group (WG) questions. The Washington Group on Disability Statistics was originally established in 2001 as a United Nations Statistical Commission Group to improve and standardise disability measurement in national surveys and censuses [8]. The WG questions capture self-reported activity limitations in functional domains described in the International Classification of Functioning, Disability and Health (ICF); for example, reported difficulties seeing, hearing and with mobility [9]. The questions purposefully focus on activity limitations rather than impairments or participation restrictions. Activity limitations are perceived to be universal, allowing for comparable data collection across settings [10]. Conversely, measurements of impairments would require clinical expertise and equipment beyond the scope of many surveys, and participation restrictions may vary too much between contexts to develop universally applicable questions. The WG questions have been used in over 100 national censuses to date [11]. They are recommended by numerous United Nations agencies and international disability advocates, both as a tool to disaggregate the Sustainable Development Goal (SDG) Indicators, and to monitor implementation of the United Nations Convention on the Rights of People with Disabilities (UNCRPD) [11,12].

There are several different WG modules recommended for the adult or all-age (5+) populations, as summarised in Table 1. In addition, and not the subject of this paper, the WG/UNICEF Child Functioning Module has been developed to capture data on children aged 2 to 17 [13]. The modules range in breadth from the 6 functional domains captured in the Short Set (SS) to the 11 captured in the Extended Set (ESF). The SS was developed primarily for use in censuses and thus focuses on a subset of “core” functional domains from the ICF that are anticipated to identify “the majority but not all” persons with disabilities and to represent the most commonly occurring limitations [14]. Notably, the SS does not include psychosocial domains, which are instead captured in longer modules designed for population-based surveys where resources allow. There are other differences. The ESF contains a number of optional questions, allowing users to capture more detail in certain domains. For example, while the SS contains one question each on seeing and mobility respectively, the ESF includes difficulty seeing at a distance or nearby, and difficulty walking both a long and a short distance. Additionally, some domains include multiple items. There are also hybrid options: the Labor Force Survey Disability Module (LFS-DM, referred to in some studies as the Modified Extended Set) includes eight domains (short set, plus anxiety and depression), and the Short Set Enhanced (SS-E) adds two further upper-body functioning questions. Respondents are asked separately whether they use common assistive products (glasses, hearing aids or mobility support) in the ESF, but this is embedded into the relevant question in the SS (“do you have difficulty seeing, even when wearing your glasses”). According to the tool’s developers, the estimated time to administer each module per respondent ranges from 1.5 min for the SS, to 10–12 min for the ESF [15].

In terms of analysis, the World Report on Disability used Item Response Theory and Rasch modelling techniques to ascertain an *a posteriori* binary disability threshold cut-off based on the distribution of observed data in the World Health Surveys. In contrast, the WG questions use *a priori* cut-offs to determine the proportion of the population “at risk of restricted participation” based on their response on a rating scale (predominantly a four-point scale per question of “no difficulty”, “some difficulty”, “a lot of difficulty” or “unable to do”) [16]. The standard pre-determined threshold recommended for calculating internationally comparable disability prevalence data is to include anyone reporting “any domain a lot of difficulty or cannot do”, but response options differ for several domains (anxiety, depression, pain and fatigue), and a wider threshold (some difficulty or worse) is often reported alongside, or in place of, the standard threshold in the literature [17].

Population-based disability estimates derived using the WG tools can vary considerably and are frequently lower than 15% [18]. In part, this will be related to contextual differences between settings that impact on prevalence—for example, cultural understandings and reporting of functional limitations, population demographics or access to health or rehabilitative services [6,19]. From a methodological perspective, differences may also be related to variability in the choice of WG module in different data collection activities, the sub-group of the population included (e.g., whether children or institutionalised populations are sampled) and the threshold set for calculating disability prevalence, all of which directly impact on outputs by capturing a greater or lesser proportion of the population experiencing functional limitations [17,20]. There may also be differences in the application of the module between surveys, namely, whether an individual self-reports or is reported for by a proxy; or the translation of questions and concepts across multiple indigenous languages.

The availability and international comparability of disability statistics has greatly improved with the adoption and use of the WG tools. However, variability in their application and in users’ comprehension of nuanced differences between modules and in comparison to other approaches remain. A recently published report by Mitra and Yap (2021) includes prevalence outputs using the WG SS or other functional limitation tools from 41 countries [21]. The report describes wide variation in estimates between countries and uses a wider threshold of “any difficulty functioning” (i.e., including those reporting “some” or greater difficulty) to estimate a median functional difficulty prevalence of 12.6% across studies. However, the report does not explore the heterogeneity of methodologies across the studies or the implications of these on outputs and their interpretation.

In this paper, we aim to compare disability prevalence estimates by WG module, threshold, application and domain and assess whether alternative combinations of questions may be valuable. We approach this through secondary analyses of seven population-based surveys in Low and Middle-Income Countries (LMICs) using the WG tools completed between 2013–2019.

## 2. Materials and Methods

### 2.1. Population-Based Surveys

We used data from cross-sectional population-based surveys using similar methods and sampling designs and completed by this manuscript’s authorship group since 2013 in seven LMICs: Cameroon (North-West Province, 2013), Guatemala (National, 2016), India (Mahbubnagar district, Telangana State, 2014), Maldives (National, 2017), Nepal (Tanahun District, 2016), Turkey (Syrian Refugee population in Sultanbeyli district, Istanbul, 2019) and Vanuatu (SANMA and TORBA provinces, 2019). The methods and sample sizes for each survey are summarised in Table 2. All surveys included children, but we have restricted our analyses to adults 18 years and older because a separate tool developed by the Washington Group and UNICEF is recommended for children [22].

The datasets include data from five of the six WHO regions and include two nationally representative samples. In Vanuatu, a population census was completed of all eligible participants across the two included provinces [23]. In Turkey, the sample was selected from one population sub-group only (Syrian refugees) using the municipality refugee database as the sampling frame [24]. All remaining surveys used two-stage cluster-based sampling from the most recent census, with clusters first selected with probability proportionate to size, and modified compact segment sampling within these to reach the desired cluster size [20,23,25,26].

### 2.2. Measurement of Disability

All seven surveys used the Washington Group modules to estimate disability prevalence. The Cameroon, India and Guatemala surveys used the ESF (11 domains, optional questions not included); the Maldives, Nepal and Turkey used the SS-E (9 domains); and Vanuatu used the LFS-DM (8 domains). All surveys prioritised self-report and allowed proxy respondents where the participant was not able to communicate independently. Additional protocols allowing for the use of proxies if the participant was unavailable were included in Guatemala, Maldives, Nepal and Vanuatu only. In other settings, unavailable participants were recorded as such, and WG data was not collected for them. All surveys except Vanuatu asked about the use of assistive products or personal assistance as separate questions before asking about difficulty with the corresponding domains with or without products/assistance as appropriate. Vanuatu used the short-set style of questions for vision and hearing in which assistive product use is embedded (e.g., do you have difficulty seeing, even when wearing your glasses). All surveys except Turkey and Vanuatu included an additional question on the use of mobility products or personal assistance for mobility for completeness, which is usually only used in the ESF. Questionnaires are provided in Table S1.

### 2.3. Data Analysis and Sample Adjustment

Data analyses for Cameroon, India, Guatemala and Vanuatu were completed using the R *survey* package [27]. Analyses for the Maldives, Nepal and Turkey were completed in STATA 16.0, using the *svyset* command to account for sampling procedures [28]. Survey samples were self-weighting by age and sex in all settings, except Cameroon and the Maldives. Consequently, age-sex and cluster-adjusted estimates are provided for Cameroon and the Maldives, whereas cluster-only-adjusted estimates are provided for other surveys.

The *ggpubr* package in R was used to generate bubble plots depicting the proportion of the sample who met the threshold for estimating prevalence by domain in each survey, for both the standard (“a lot of difficulty” or worse) and wide thresholds (“some difficulty” or worse). Bubble circumference represents crude sample prevalence for each domain. The inner circle of each bubble represents the additional percentage point prevalence derived from each domain (i.e., “novel” participants added to the prevalence estimate), whereas the outer band represents the percentage points already identified via other domains (i.e., participant identified via multiple domains). Comparisons are stepwise by module, i.e., we added percentage point prevalence among the SS domains, then new domains of LFS-DM, SS-E and ESF, respectively.

## 3. Results

The sample size of adults aged 18+ in the surveys ranged from 1554 in Turkey to 31,362 in Vanuatu (Table 2). The average age of the 18+ sub-samples used in these analyses ranged between 34.8 years in Turkey and 44.5 years in Cameroon, and the response rate ranged from 77% in Turkey to 95% in Nepal. The completion of data collection by proxy varied from 0% in Cameroon, India and Turkey to 24.7% in Vanuatu.

Overall, the prevalence of disability in the population aged 18+ tended to increase as modules were included with an additional number of items (Table 3). Based on the SS, the prevalence of disability ranged from 3.2% (95% Confidence Interval 2.9–3.6) in Vanuatu to 14.1% (12.2–16.2) among Syrian Refugees in Turkey. Including the additional domains of anxiety and depression (LFS-DM), the point prevalence increased in all settings compared with the SS and was statistically higher in Guatemala (9.1%, 8.3–10.0), Turkey (21.3%, 18.3–24.6) and Vanuatu (6.3%, 5.5–7.2). There were no significant differences in prevalence using the SS-E (additional domain of upper body strength) compared with the LFS-DM in any survey. The estimate using the ESF was significantly higher than the SS in the three countries that used this question set (Cameroon 12.9% (11.0–14.9), Guatemala 11.1% (10.2–12.1) and India 14.7% (12.5–17.2)), but not significantly higher than the LFS-DM (India) or SS-E (Guatemala).

Using a wider threshold of “some” or greater functional limitations substantially increased the estimated prevalence of disability using each module, often by between 5- and 10-fold (Table 4). For example, the SS prevalence in Cameroon increased from 6.1% (4.5–7.9) using the standard definition to 66.3% (63.2–69.4) using the wide threshold. However, at a wider threshold, there was a less clear increase in disability prevalence as WG modules with more items were used.

**Figure 1:**
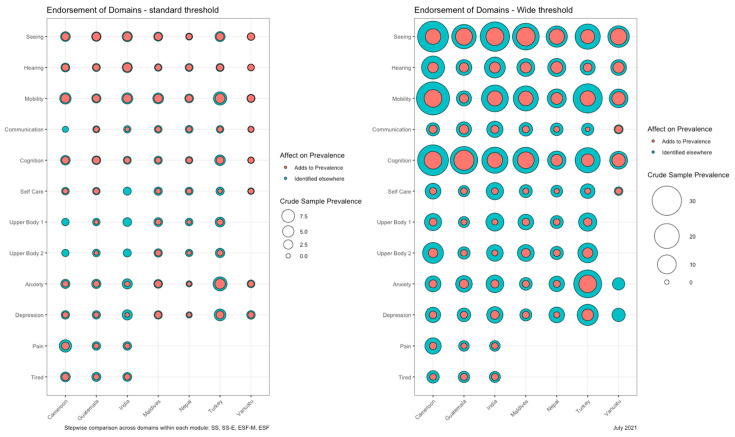
Reported difficulties in domains using standard and wide thresholds.

Figure 1 depicts the crude sample prevalence of limitations in each domain per survey, using both the standard and wide thresholds. At the standard threshold, *mobility*, *seeing, hearing* and *cognition* were the most commonly reported SS domains across surveys (accounting for a combined population point prevalence of between 1.3% in Nepal and 5.4% in India), with limited overlap between them. The *communication* and *self-care* domains identified the fewest participants overall, and the fewest novel participants not already identified via any other domains (between not increasing the overall prevalence at all in India and increasing it by 0.2 percentage points in Guatemala). The two additional questions related to the *upper body* domain (SS-E) identified no additional participants in Cameroon or India and few additional participants in other settings (maximum 0.4 percentage points in Turkey). Some participants identified via the *anxiety* and *depression* domains were identified elsewhere, but the prevalence of these domains was high across studies and identified particularly high numbers of additional participants in Turkey (4.9 percentage points combined across the two domains). Adding the two additional ESF domains in the three surveys that used this module, *pain* and *fatigue* combined added between 0.3 and 1.9 percentage points to study prevalence estimates.

Using the wider threshold, there was substantially greater overlap between the reported difficulties with domains in all surveys. The prevalence of functional limitations related to *seeing*, *hearing* and *mobility* were still high compared with other domains (up to 39.8% reporting “some” or greater difficulty with *mobility* in Cameroon and 30.4% reporting “some” or greater difficulty *seeing* in India). However, approximately two-thirds of responders in each dataset reported difficulties with additional domains as well. The proportion of the population reporting “some” or greater difficulty with *cognition* was much higher than “a lot” of difficulty in each survey—ranging between 8.7% in Vanuatu (of which 3.6% were novel) and 33.8% in Cameroon (of which 8.3% were novel). The proportion of the population reporting wider thresholds of *anxiety—*up to 26.7% in Turkey and 8.5% in India—and *depression*—13.3% in Turkey and 6.8% in India—was variable. The reported difficulties were higher for all remaining domains than at the standard threshold, however, few novel participants were included in each domain as a result.

Alternative combinations of items to existing modules may come closer to the estimated prevalence from the full ESF. Replacing the *communication* and *self-care* domains in the short set with the two affect domains, would generate prevalence estimates of between 8.1% (6.4–10.0) in Cameroon and 13.1% (10.9–15.5) in India (Table 5). For 8 items, the combination of *seeing*, *hearing, mobility, cognition, anxiety, depression, pain* and *fatigue*, produces disability prevalence estimates of 13.0% (11.1–15.1) in Cameroon, 10.8% (9.9 0 11.8) in Guatemala and 14.5% (12.3–16.9) in India (combination not available for other datasets).

We disaggregated the estimates of limitations *seeing*, *hearing* and *mobility* by assistive product/support use in the six surveys that collected this separately (Supplementary Material Table S2. Broadly, we found that the proportion of the population who reported “some” or greater difficulty *seeing* was higher among those who wore glasses (while wearing them) compared to those who didn’t in several surveys, but this trend was not observed at a higher threshold of “a lot” of difficulty. Among people who reported using mobility equipment or the support or another person, the majority reported some or greater difficulty with *mobility* even while using equipment/support, which was much higher than among people who did not report using mobility equipment/support. The reported use of hearing aids was too low to complete any further analyses.

## 4. Discussion

### 4.1. Key Findings

The prevalence estimates from the 7 surveys ranged considerably from 3.2% (2.9–3.6) in Vanuatu to 14.1% (12.2–16.2) in Turkey, using the Short Set (SS) standard threshold, and between 11.1% (10.2–12.1) in Guatemala and 14.7% (12.5–17.2) in India using the Extended Set (ESF) standard threshold. Using a wider threshold resulted in between a 5- and 10-fold increase in the proportion of the population included in the estimate and diminished the differences between estimates from different modules. Certain domains captured more “new” participants with functional limitations (i.e., who were not captured by any other domain) than others and people who reported wearing glasses or using mobility products/assistance were more likely to report “some” or “a lot” (mobility only) of difficulty with the corresponding domain, even when using their assistive devices/support.

### 4.2. Accounting for Variability in Prevalence Estimates

Our analyses highlight the variability in estimates generated using the WG tools in different settings, even when deployed in a standardised way in the same population age-group. These differences emphasise both contextual and methodological factors affecting the frequency of reported functional limitations in different settings. Contextually, across the included surveys, the short set prevalence was lowest in Vanuatu, where the population is relatively young. Low disability prevalence estimates have been observed in several other Pacific Island countries with similar demographic profiles [29,30]. In contrast, the prevalence was highest among Syrian refugees in Turkey, driven largely by the high prevalence of anxiety and depression as may be expected in a conflict-affected, displaced population [23,31]. Mitra and Yap (2021) reported similar variability in outputs [21]. The authors present a median prevalence of functional limitations for adults 15 and older of 2.5% at the standard threshold, with wide variation across countries (a range from 0.8% in Vanuatu to 12.2% in Columbia). Notably, a wider threshold of “any functional limitation” is used to describe the median prevalence of 12.6% across studies in the report’s executive summary. Further highlighting the methodological nuance, the Vanuatu 2009 Census included in the report used a four-question tool with different response options to the standard SS, generating a much lower estimate than our Vanuatu study.

In terms of methodological differences, higher estimates were derived across our datasets using the SS-E and ESF compared to the SS within surveys, on account of the additional domains captured in the latter tools. Considering the spectrum of functioning and functional limitations as described in the ICF, this is to be expected. In particular, the inclusion of *anxiety*, *depression*, *pain* and *fatigue* identified a greater proportion of new participants—who would otherwise be excluded from prevalence estimates—compared to two of the short set domains (*communication* and *self-care*). This underlines the advantage of utilising the expanded modules where possible and determining the added-value of specific domains—particularly those that do not “add” to the prevalence estimates. Additionally, it brings into question whether the current SS includes the most pertinent domains to capture the majority of persons with disabilities, as it intends. In particular, mental health conditions are not only common, but can also be more stigmatising than some SS domains, resulting in greater barriers to participation or implications on wellbeing [32,33]. Similarly, chronic pain or fatigue are largely absent from the discourse around disability inclusion, despite evidence of their high prevalence and association with both functional limitations and participation restrictions in LMICs [34,35].

Estimates using the wider threshold for each module were substantially higher across all surveys compared to the standard threshold (maximum 71.0%, 67.7–74.2 for the ESF in Cameroon). We have previously reported positive correlation between reporting “some” difficulty and having mild or worse clinical impairments benefiting from intervention in respective domains, and mixed findings on the relationship between reporting “some” difficulty and reporting participation restrictions [20,23]. Considering the high proportion of each sample reporting “some” or greater difficulty across domains who may potentially benefit from support or accommodation, presentation of this threshold (potentially described as the population *at risk* of disability) alongside the standard estimate may be valuable.

We disaggregated outputs for people who did, and did not, report using assistive products in the datasets that asked this separately. These findings identified a trend towards increased use of assistive devices/support correlating with increased prevalence estimates in the relevant domain. Few people accessed hearing aids but for vision and mobility, this may relate to the availability of poor-quality assistive products that do not improve functioning for people with difficulties in the relevant domain. As the mobility question includes “support”, and given that the coverage of appropriate, locally manufactured assistive products for mobility support is generally low across LMICs, this may also reflect a mix of manufactured products, non-standard products and informal support from family or community members [36,37]. Further work is needed to understand how the reported use of assistive products affects prevalence estimates, particularly in the short set where this is embedded into the relevant question for the *seeing* and *hearing* domains. Given variability in usage and the need for better data on assistive device use in LMICs, it may be preferable to disentangle assistive device use from the SS and report the use of devices separately [37].

Our results potentially flag differences related to application of the modules, too. The lowest prevalence estimates were found in Vanuatu and Nepal where there were higher proportions of proxy reporting, while higher estimates were found in those surveys that used self-report only. Similarly, a recent secondary analysis of three Demographic and Health Survey datasets in South Africa, Uganda and Mali identified significant under-reporting of functional limitations by proxies, particularly in non-observable domains [38]. The same study also suggested that communication difficulties may be over-reported in comparison to self-report. These discrepancies will affect prevalence outputs and may be important, given the tendency for many household surveys and censuses to rely on household head reports, which may therefore lead to underestimates of prevalence [39,40].

### 4.3. Reflecting on 15% and Disability Data Collection Approaches in Population-Based Surveys

Across the different studies in our analyses, the ESF estimates using the standard threshold provided the closest estimates to the World Report’s 15%. It is important to acknowledge the divergence in approaches underlying these estimates, and that the 15% estimate does not represent a “ground truth” or gold standard to which newer outputs should be compared. To arrive at 15% in the World Report, complex statistical techniques were used to transform the WHS composite scores across domains into a cumulative distribution, before a post hoc binary threshold for “disabled” was applied, based on the average score of respondents reporting a range of health conditions (e.g., asthma) commonly associated with functional limitation [4]. Such an *a posteriori* classification of disability based on response distribution is also used in the WHO’s Model Disability Survey (MDS) [41]. This approach provides a comprehensive description of the continuum of functioning across a given population, reflecting the contextual factors in that population’s respective environment. However, it requires considerable statistical competency and does not allow for the application of classifications during data collection that may be useful (for example, to collect more detailed data on those who meet the disability threshold or to refer those with unmet needs to services).

Mitra (2017) converted WG responses in four datasets into a composite “functional score”, based on respondents’ combined score across the six SS questions. This numeric score was then regressed against various indicators of individual and household wellbeing, such as work and food insecurity, identifying associations between multidimensional poverty and functioning [17]. Disability prevalence in the study was estimated using the standard threshold approach, rather than by using the “functional score” to model an alternative that was aligned with the WHO approach. 

Alternatively, in the UK and Europe, where the definition of disability in legislation includes participation restrictions, short tools such as the UK Equality Act Disability Definition (EADD) and the Global Activity Limitation Instrument (GALI) are common in household surveys. These tools provide outputs on the prevalence of long-term health conditions that reduce respondents’ “ability to carry out day to day activities” [42]. A recent UK study estimated 28% prevalence of disability using the EADD, compared with 9% and 13% using the standard thresholds for the SS and ESF, respectively [42]. However, such tools do not provide data on specific functional limitations which may be useful for planning and prioritising need, and do not capture whether the use of assistive products is included in the individual’s response.

### 4.4. Implications for Disability Prevalence Measurement

Considerable efforts and advances have been made by stakeholders to harmonise population-based disability data collection using the WG tools, which are simple to use and analyse, aligned with the UNCRPD and the ICF and appropriate for disaggregation of the SDGs. These achievements are laudable and have greatly progressed the feasibility and comparability of disability data collection, particularly in LMICs. However, there are numerous differentiators among the WG modules and in their use that affect outputs derived from them. It is important that collectors and users of disability data understand what the WG tools are capturing, the implication of using different modules and different applications, and what is missed. Additionally, that the methodological approach using the WGs is divergent from the modelling used to generate the often-quoted 15% global prevalence of disability and will consequently generate dissimilar outputs [18].

Importantly, our findings suggest that the current set of domains in the SS may be sub-optimal in identifying the majority of people with functional limitations at risk of participation restrictions. In particular, our findings suggest that the *communication* and *self-care* domains are of limited value in identifying additional members of the population with limitations not already identified elsewhere. Instead, a preferable six-item set to capture the majority of functional limitations without including redundant or optional questions may be *seeing*, *hearing, mobility, cognition, anxiety* and *depression,* ideally with separate questions (not integrated) to capture use of products for *vision*, *hearing* and *mobility*, respectively. In circumstances where resources allow, increasing this to eight items and including *pain* and *fatigue* would identify close to the proportion identified in the ESF but with 50% fewer items overall. The testing of these combinations, including the time each suggested question set would take to administer, would be of value. Further formal assessment of the implications of proxy report for the WG tools is also needed, disaggregated by domain. Moreover, the proportion of data completed by proxy report and the proportion of the population included using the wider threshold should also be presented as standard.

Updated global estimates using the WG tools are perhaps also merited, considering the divergence in methodologies since the World Report was published, the variation in estimates produced using the WG tools and shifting demographics. An *a posteriori* analysis of available WG data, triangulated with reported participation restrictions where available, may support this study and shed further light on optimum cut-offs and whether these vary by context.

### 4.5. Strengths and Limitations

Over 53,000 adults from 7 LMICs are included in these secondary analyses, providing rich comparative data on disability prevalence using the WG tools. Through disaggregating estimates by threshold, module and domain, we have been able to systematically interrogate and reflect on the outputs. However, our analyses are restricted to the adult population in each setting. In addition, while we estimated the effects of assistive products, we did not address other contextual factors, or the association between reported limitations and impairments or participation restriction. Finally, the data available did not allow us to attempt to replicate Rasch modelling techniques and compare outputs using this approach.

## 5. Conclusions

The Washington Group tools are extremely valuable in promoting harmonised population-based data collection on disability. It is important that collectors and users of disability data using the WG tools are aware of the implications of the use of different modules and thresholds and recognise the added value of different domains. We recommend that alternate combinations of domains (6-item: *seeing*, *hearing*, *mobility*, *cognition*, *anxiety*, *depression*; 8-item: plus *pain*, *fatigue*) are tested to capture a greater proportion of people with functional limitations without substantially increasing the module length. Additionally, we recommend that the following is reported separately as standard: a “wide” threshold, not conflated with disability prevalence; the proportion of responses completed by proxy; and the proportion of the population using assistive products.

## Figures and Tables

**Table 1:** Washington Group modules and thresholds.

Module	Items	Response Options	Threshold-Standard	Threshold-Wide
Short Set (SS)	Do you have difficulty in seeing, even if wearing glasses? Do you have difficulty in hearing, even if using a hearing aid? Do you have difficulty walking or climbing steps? Do you have difficulty remembering or concentrating? Do you have difficulty with self-care, such as washing all over or dressing? Using your usual language, do you have difficulty communicating, for example understanding or being understood?	No difficulty Some difficulty A lot of difficulty Cannot do at all	Any domain a lot of difficulty *or* unable to do	Any domain some difficulty
Labor Force Survey Disability Module (LFS-DM)	Short Set as above, plus anxiety and depression questions detailed below: 7. How often do you feel worried, nervous or anxious? 8. Thinking about the last time you felt worried, nervous or anxious, how would you describe the level of these feelings? 9. How often do you feel depressed? 10. Thinking about the last time you felt depressed, how depressed did you feel?	Questions 7 and 9: Daily Weekly Monthly A few times a year Never Questions 8 and 10: A little A lot Somewhere between a little and a lot	Either domain daily *and* a lot	Either domain daily or weekly *and* a lot, or in between a little and a lot
Short Set Enhanced (SS-E)	Labor Force Survey Disability Module as above, plus upper body function questions detailed below: 11. Do you have difficulty raising a 2-litre bottle of water or soda from waist to eye level? 12. Do you have difficulty using your hands and fingers, such as picking up small objects, for example, a button or pencil, or opening or closing containers or bottles?	No difficulty Some difficulty A lot of difficulty Cannot do at all	Either domain a lot of difficulty *or* unable to do	Any domain some difficulty
Extended Set on Functioning (ESF)	Short Set Enhanced as above, plus pain and fatigue questions detailed below: 13. In the past 3 months, how often did you have pain? 14. Thinking about the last time you had pain, how much pain did you have? 15. In the past 3 months, how often did you feel very tired or exhausted? 16. Thinking about the last time you felt very tired or exhausted, how long did it last? 17. Thinking about the last time you felt this way, how would you describe the level of tiredness? In addition: use of assistive products was asked as separate questions, and respondents were asked whether they have difficulties with, domain with, and separately without, their products	Questions 13 and 15: Never Some days Most days Every day Questions 14 and 17: A little A lot Somewhere between a little and a lot Question 16: Some of the day Most of the day All of the day	Pain: Every day *and* a lot Fatigue: Most days *and* all of the day *or* Every day *and* most of the day *or* Every day *and* all of the day	No change

**Table 2:** Data sources (18+).

WHO Region ˆ	AFRO	AMRO	SEARO	SEARO	SEARO	EURO	WRPO
Dataset name (Country of origin)	Cameroon	Guatemala	India	Maldives	Nepal	Turkey	Vanuatu
Place, date	Fundong Health District (North West), 2013	National, 2016	Mahbubnagar District, Telengana State, 2014	National, 2017	Tanahun District, 2016	Sultanbeyli district Istanbul, Turkey 2019	SANMA and TORBA province, 2019
Sampling Strategy	Two stage Cluster Sampling, Clusters of 80 ^‡^	Two stage Cluster Sampling, Clusters of 50 ^‡^	Two stage Cluster Sampling, Clusters of 80 ^‡^	Two stage Cluster Sampling, Clusters of 125 ^‡^	Two stage Cluster Sampling, Clusters of 200 ^‡^	Syrian refugees only: 80 clusters of 50 people selected from the municipality refugee database with probability proportionate to size	Full population census
WG modules	ESF	ESF	ESF	SS-E	SS-E	SS-E	LFS-DM Anxiety and depression questions only asked if self-report, not proxy
WG respondent	Self unless unable to communicate, no proxy if unavailable	Self unless unable to communicate or if unavailable after 2 visits	Self unless unable to communicate, no proxy if unavailable	Self unless unable to communicate or if unavailable after 3 or more attempts	Self unless unable to communicate or if unavailable after 3 or more attempts	Self unless unable to communicate, no proxy if unavailable	Self unless unable to communicate or if unavailable (after 2 attempts where feasible)
% WG completed by proxy	0	8.5%	0	36.8%	6.4%	0	24.7%
Assistive products	Use of glasses, hearing aids and mobility products reported separately	Use of glasses, hearing aids and mobility products reported separately	Use of glasses, hearing aids and mobility products reported separately	Use of glasses, hearing aids and mobility products reported separately	Use of glasses, hearing aids and mobility products reported separately	Use of glasses and hearing aids reported separately	Included within vision and hearing question
Total Sample (response rate%)	3567 (87%)	13,073 (88%)	3574 (88%)	5362 (82%)	5692 (95%)	3084 (77%)	56,402 (85%)
Sub-sample size (complete data%)	1617 (96%)	8910 (85%)	2350 (99%)	3592 (100%)	4067 (100%)	1554 (99.7%)	31,362 (100%)
Average age (years), range	44.5 (18–99)	38.7 (18–100)	39.2 (18–98)	39.4 (18–102)	43.0 (18–96)	34.8 (18–90)	37.7 (18–115)
% Female	70%^†^	54%	54%	58.0%^†^	57.7%	56.3%	49%

ˆ WHO Regional Abbreviations: Africa (AFRO), Americas (AMRO), Eastern Mediterranean (EMRO), Europe (EURO), South-East Asia (SEARO), Western Pacific (WPRO) ^‡^ In each case, this included selection of enumeration areas (clusters) from most recent Census with Probability Proportionate to Size (PPS), followed by modified Compact Segment Sampling (CSS) within clusters until pre-determined number of participants per cluster enumerated without replacement. ^†^Sample substantially different to census, age-sex adjusted estimates presented.

**Table 3:** Disability prevalence by module type—Standard threshold.

Module	Cameroon (*n* = 1617)	Guatemala (*n* = 8910)	India (*n* = 2350)	Nepal (*n* = 4067)	Maldives (*n* = 3592)	Turkey (*n* = 1554)	Vanuatu (*n* = 31,362)
	% (95% CI)	% (95% CI)	% (95% CI)	% (95% CI)	% (95% CI)	% (95% CI)	% (95% CI)
Short Set (6 items)	6.1 (4.5–7.9)	7.3 (6.6–8.0)	9.8 (7.7–12.1)	4.1 (3.6–4.8)	6.4 (5.7–7.2)	14.1 (12.2–16.2)	3.2 (2.9–3.6)
Labor Force Survey (10 items)	8.0 (6.4–9.9)	9.1 (8.3–10.0)	13.2 (11.1–15.6)	4.2 (3.6–4.9)	8.0 (7.2–8.9)	21.3 (18.3–24.6)	6.3 (5.5–7.2)
Short Set Enhanced (12 items)	8.0 (6.4–9.9)	9.3 (8.5–10.2)	13.2 (11.1–15.6)	4.7 (4.1–5.4)	8.4 (7.6–9.4)	21.7 (18.7–25.0)	-
Full ESF (17 items)	12.9 (11.0–14.9)	11.1 (10.2–12.1)	14.7 (12.5–17.2)	-	-	-	-

**Table 4:** Disability prevalence by module type—Wider threshold.

Module	Cameroon (*n* = 1617)	Guatemala (*n* = 8910)	India (*n* = 2350)	Nepal (n = 4067)	Maldives (*n* = 3592)	Turkey (*n* = 1554)	Vanuatu (*n* = 31,362)
	% (95% CI)	% (95% CI)	% (95% CI)	% (95% CI)	% (95% CI)	% (95% CI)	% (95% CI)
Short Set (6 items)	66.3 (63.2–69.4)	50.4 (48.8–52)	56.0 (52.6–59.3)	26.5 (25.2–27.9)	46.5 (51.9–55.2)	48.5 (44.8–52.2)	29 (27.4–30.6)
Labor Force Survey (10 items)	68.5 (65.2–71.7)	52.3 (50.6–54)	57.7 (54.3–61.1)	26.9 (25.6–28.3)	47.3 (45.7–49.0)	56.5 (52.1–61.0)	35.6 (33.9–37.3)
Short Set Enhanced (12 items)	70.3 (66.9–73.6)	52.8 (51.1–54.4)	57.9 (54.5–61.3)	27.6 (26.2–29.0)	48.7 (47.0–50.4)	59.0 (54.6–63.2)	-
Full ESF (17 items)	71.0 (67.7–74.2)	53.2 (51.5–54.9)	58.4 (55.1–61.8)	-	-	-	-

**Table 5:** Disability prevalence by alternative question sets—Standard threshold.

Domains	Cameroon (*n* = 1617)	Guatemala (*n* = 8910)	India (*n* = 2350)	Nepal (*n* = 4067)	Maldives (*n* = 3592)	Turkey (*n* = 1554)
	% (95% CI)	% (95% CI)	% (95% CI)	% (95% CI)	% (95% CI)	% (95% CI)
6 Items ^1^	8.1 (6.4–10.0)	8.9 (8.1–9.8)	13.1 (10.9–15.5)	3.9 (3.3–4.5)	7.9 (7.1–8.8)	21.2 (18.2–24.5)
8 Items ^2^	13.0 (11.1–15.1)	10.8 (9.9–11.8)	14.5 (12.3–16.9)	-	-	-

^1^ Seeing, Hearing, Mobility, Cognition, Anxiety, Depression. ^2^ Seeing, Hearing, Mobility, Cognition, Anxiety, Depression, Pain and Fatigue.

## Data Availability

We are unable to make the databases publicly available as we did not have participant consent for this. We can, however, share the databases with researchers upon request.

## Supplementary Materials

The following are available online at https://www.mdpi.com/article/10.3390/ijerph18179213/s1, Table S1: Washington Group Tools as used in each of the included surveys, Table S2: Prevalence of functional limitations among subpopulations with and without assistive products.
