# Supplementary material for: Interrogating and Reflecting on Disability Prevalence Data Collected Using the Washington Group Tools: Results from Population-Based Surveys in Cameroon, Guatemala, India, Maldives, Nepal, Turkey and Vanuatu
Source: Int J Environ Res Public Health. 2021 Aug 31;18(17):9213. doi: 10.3390/ijerph18179213 (PMC8431177; doi:10.3390/ijerph18179213)
Supplement: Supplementary file 1 [file ijerph-18-09213-s001.zip › ijerph-1339871-supplementary.pdf]

Table S1. Washington Group Tools as used in each of the included surveys.

NB excludes optional questions included in survey but not in analyses

1A. Extended Set on Functioning (ESF) as used in Cameroon, India and Guatemala

#	Question	Response options
VIS_1	[Do/Does] [you/he/she] wear glasses or contact lenses?	Yes, No
VIS_2	[Do/Does] [you/he/she] have difficulty seeing, [If VIS_1 = 1: even when wearing [your/his/her] glasses/contact lenses ?	No difficulty, some difficulty, a lot of difficulty, cannot do
HEAR_1	[Do/Does] [you/he/she] use a hearing aid?	Yes, No
HEAR_2	[Do/Does] [you/he/she] have difficulty hearing, [If HEAR_1 = 1: even when using a hearing aid(s)]?	No difficulty, some difficulty, a lot of difficulty, cannot do
MOB_1	[Do/Does] [you/he/she] have difficulty walking or climbing steps?	No difficulty, some difficulty, a lot of difficulty, cannot do
MOB_2	[Do/does] [you/he/she] use any equipment or receive help for getting around?	Yes, No
MOB_MOD	[if MOB_2 = 1] [Do/does] [you/he/she] have difficulty walking or climbing steps, even when using your equipment or with help?	No difficulty, some difficulty, a lot of difficulty, cannot do
COG_SS	[Do/does] [you/he/she] have difficulty remembering or concentrating?	No difficulty, some difficulty, a lot of difficulty, cannot do
SC_SS	[Do/does] [you/he/she] have difficulty with self-care, such as washing all over or dressing?	No difficulty, some difficulty, a lot of difficulty, cannot do
COM_SS	Using [your/his/her] usual language, [do/does] [you/he/she] have difficulty communicating, for example understanding or being understood?	No difficulty, some difficulty, a lot of difficulty, cannot do
UB_1	[Do/Does] [you/he/she] have difficulty raising a 2 liter bottle of water or soda from waist to eye level?	No difficulty, some difficulty, a lot of difficulty, cannot do
UB_2	[Do/Does] [you/he/she] have difficulty using [your/his/her] hands and fingers, such as picking up small objects, for example, a button or pencil, or opening or closing containers or bottles?	No difficulty, some difficulty, a lot of difficulty, cannot do
ANX_1	How often [do/does] [you/he/she] feel worried, nervous or anxious?	Daily, weekly, monthly, a few times a year, never
ANX_2	Thinking about the last time [you/he/she] felt worried, nervous or anxious, how would [you/he/she] describe the level of these feelings?	A little, a lot, somewhere between a little and a lot
DEP_1	How often [do/does] [you/he/she] feel depressed?	Daily, weekly, monthly, a few times a year, never
DEP_2	Thinking about the last time [you/he/she] felt depressed, how depressed did [you/he/she] feel?	A little, a lot, somewhere between a little and a lot
PAIN_1	In the past 3 months, how often did [you/he/she] have pain?	Never, some days, most days, every day
PAIN_2	Thinking about the last time [you/he/she] had pain, how much pain did [you/he/she] have?	A little, a lot, somewhere between a little and a lot
TIRED_1	In the past 3 months, how often did [you/he/she] feel very tired or exhausted?	Never, some days, most days, every day
TIRED_2	Thinking about the last time [you/he/she] felt very tired or exhausted, how long did it last?	Some of the day, most of the day, all of the day
TIRED_3	Thinking about the last time [you/he/she] felt this way, how would you describe the level of tiredness?	A little, a lot, somewhere between a little and a lot

1B Short Set Enhanced (SS-E) as used in Nepal and Maldives

#	Question	Response options
VIS_1	[Do/Does] [you/he/she] wear glasses or contact lenses?	Yes, No
VIS_2	[Do/Does] [you/he/she] have difficulty seeing, [If VIS_1 = 1: even when wearing [your/his/her] glasses/contact lenses ?	No difficulty, some difficulty, a lot of difficulty, cannot do
HEAR_1	[Do/Does] [you/he/she] use a hearing aid?	Yes, No
HEAR_2	[Do/Does] [you/he/she] have difficulty hearing, [If HEAR_1 = 1: even when using a hearing aid(s)]?	No difficulty, some difficulty, a lot of difficulty, cannot do

MOB_1	[Do/Does] [you/he/she] have difficulty walking or climbing steps?	No difficulty, some difficulty, a lot of difficulty, cannot do
MOB_2	[Do/does] [you/he/she] use any equipment or receive help for getting around?	Yes, No
MOB_ MOD	[if MOB_2 = 1] [Do/does] [you/he/she] have difficulty walking or climbing steps, even when using your equipment or with help?	No difficulty, some difficulty, a lot of difficulty, cannot do
COG_SS	[Do/does] [you/he/she] have difficulty remembering or concentrating?	No difficulty, some difficulty, a lot of difficulty, cannot do
SC_SS	[Do/does] [you/he/she] have difficulty with self-care, such as washing all over or dressing?	No difficulty, some difficulty, a lot of difficulty, cannot do
COM_SS	Using [your/his/her] usual language, [do/does] [you/he/she] have difficulty communicating, for example understanding or being understood?	No difficulty, some difficulty, a lot of difficulty, cannot do
UB_1	[Do/Does] [you/he/she] have difficulty raising a 2 liter bottle of water or soda from waist to eye level?	No difficulty, some difficulty, a lot of difficulty, cannot do
UB_2	[Do/Does] [you/he/she] have difficulty using [your/his/her] hands and fingers, such as picking up small objects, for example, a button or pencil, or opening or closing containers or bottles?	No difficulty, some difficulty, a lot of difficulty, cannot do
ANX_1	How often [do/does] [you/he/she] feel worried, nervous or anxious?	Daily, weekly, monthly, a few times a year, never
ANX_2	Thinking about the last time [you/he/she] felt worried, nervous or anxious, how would [you/he/she] describe the level of these feelings?	A little, a lot, somewhere between a little and a lot
DEP_1	How often [do/does] [you/he/she] feel depressed?	Daily, weekly, monthly, a few times a year, never
DEP_2	Thinking about the last time [you/he/she] felt depressed, how depressed did [you/he/she] feel?	A little, a lot, somewhere between a little and a lot

1C Short Set Enhanced (SS-E) as used in Turkey

#	Question	Response options
VIS_1	[Do/Does] [you/he/she] wear glasses or contact lenses?	Yes, No
VIS_2	[Do/Does] [you/he/she] have difficulty seeing, [If VIS_1 = 1: even when wearing [your/his/her] glasses/contact lenses ?	No difficulty, some difficulty, a lot of difficulty, cannot do
HEAR_1	[Do/Does] [you/he/she] use a hearing aid?	Yes, No
HEAR_2	[Do/Does] [you/he/she] have difficulty hearing, [If HEAR_1 = 1: even when using a hearing aid(s)]?	No difficulty, some difficulty, a lot of difficulty, cannot do
MOB_SS	[Do/Does] [you/he/she] have difficulty walking or climbing steps?	No difficulty, some difficulty, a lot of difficulty, cannot do
COG_SS	[Do/does] [you/he/she] have difficulty remembering or concentrating?	No difficulty, some difficulty, a lot of difficulty, cannot do
SC_SS	[Do/does] [you/he/she] have difficulty with self-care, such as washing all over or dressing?	No difficulty, some difficulty, a lot of difficulty, cannot do
COM_SS	Using [your/his/her] usual language, [do/does] [you/he/she] have difficulty communicating, for example understanding or being understood?	No difficulty, some difficulty, a lot of difficulty, cannot do
UB_1	[Do/Does] [you/he/she] have difficulty raising a 2 liter bottle of water or soda from waist to eye level?	No difficulty, some difficulty, a lot of difficulty, cannot do
UB_2	[Do/Does] [you/he/she] have difficulty using [your/his/her] hands and fingers, such as picking up small objects, for example, a button or pencil, or opening or closing containers or bottles?	No difficulty, some difficulty, a lot of difficulty, cannot do
ANX_1	How often [do/does] [you/he/she] feel worried, nervous or anxious?	Daily, weekly, monthly, a few times a year, never
ANX_2	Thinking about the last time [you/he/she] felt worried, nervous or anxious, how would [you/he/she] describe the level of these feelings?	A little, a lot, somewhere between a little and a lot
DEP_1	How often [do/does] [you/he/she] feel depressed?	Daily, weekly, monthly, a few times a year, never
DEP_2	Thinking about the last time [you/he/she] felt depressed, how depressed did [you/he/she] feel?	A little, a lot, somewhere between a little and a lot

1D. Labour Force Disability Module as used in Vanuatu

#	Question	Response options
VIS_SS	[Do/Does] [you/he/she] have difficulty seeing, even if wearing glasses?	No difficulty, some difficulty, a lot of difficulty, cannot do
HEAR_SS	[Do/Does] [you/he/she] have difficulty hearing, even if using a hearing aid(s)?	No difficulty, some difficulty, a lot of difficulty, cannot do
MOB_SS	[Do/Does] [you/he/she] have difficulty walking or climbing steps?	No difficulty, some difficulty, a lot of difficulty, cannot do
COG_SS	[Do/does] [you/he/she] have difficulty remembering or concentrating?	No difficulty, some difficulty, a lot of difficulty, cannot do
SC_SS	[Do/does] [you/he/she] have difficulty with self-care, such as washing all over or dressing?	No difficulty, some difficulty, a lot of difficulty, cannot do
COM_SS	Using [your/his/her] usual language, [do/does] [you/he/she] have difficulty communicating, for example understanding or being understood?	No difficulty, some difficulty, a lot of difficulty, cannot do
	Below questions only asked of those who self reported (not proxy)	
ANX_1	How often do you feel worried, nervous or anxious?	Daily, weekly, monthly, a few times a year, never
ANX_2	Thinking about the last time you felt worried, nervous or anxious, how would [you/he/she] describe the level of these feelings?	A little, a lot, somewhere between a little and a lot
DEP_1	How often do you feel depressed?	Daily, weekly, monthly, a few times a year, never
DEP_2	Thinking about the last time you felt depressed, how depressed did you feel?	A little, a lot, somewhere between a little and a lot

Supplementary Material: Table S2

Table S2: Prevalence of functional limitations among subpopulations with and without assistive products

	Cameroon (n=1617)	Guatemala (n=8910)	India (n=2350)	Nepal (n=4067)	Maldives (n=3592)	Turkey (n=1554)
	% (95% CI)	% (95% CI)	% (95% CI)	% (95% CI)	% (95% CI)	% (95% CI)
**Wears glasses**	**5.5 (4.1 – 6.9)**	**12.3 (11.0 – 13.5)**	**10.0 (8.1 – 11.8)**	**11.8 (10.8-12.8)**	**33.3 (31.8-34.9)**	**16.4 (14.3-18.8)**
Some or greater difficulty seeing, while wearing glasses	41.4 (27.7 – 55.1)	26.3 (23.1 – 29.6)	50.2 (42.6 – 57.9)	20.0 (16.7-23.8)	19.6 (17.5-21.9)	45.1 (37.9-52.4)
A lot or greater difficulty, while wearing glasses	1.0 (0.0 – 3.1)	4.3 (3.0 – 5.6)	3.9 (1.2 – 6.6)	0.8 (0.3-2.2)	2.2 (1.5-3.2)	7.1 (4.2-11.4)
**Does not wear glasses**	**94.5 (93.1 – 95.9)**	**87.7 (86.5 – 89.0)**	**90.0 (88.2 – 91.9)**	**88.2 (87.2-89.2)**	**66.7 (65.1-68.2)**	83.5 (81.1-85.7)
Some or greater difficulty seeing	28.2 (25.6 – 30.8)	25.7 (24.3 – 27.1)	32.5 (29.6 – 35.3)	12.8 (11.8-14.0)	24.9 (23.2-26.7)	16.3 (13.9-18.9)
A lot or greater difficulty seeing	1.5 (0.1 – 2.1)	3.0 (2.5 – 3.4)	3.6 (2.2 – 5.0)	0.6 (0.4-1.0)	2.0 (1.5-3.2)	2.7 (1.9-3.8)
**Uses mobility equipment or assistance**	**9.7 (7.9 – 11.4)**	**2.5 (2.1 – 2.9)**	**4.3 (3.3 – 5.3)**	**2.2 (1.8-2.7)**	**2.0 (1.6-2.5)**	-
Some or greater difficulty walking, using equipment/assistance	64.6 (55.5 – 73.5)	79.3 (73.6 – 84.9)	93.0 (88.2 – 97.8)	87.9 (79.4-93.2)	98.3 (88.8-99.8)	-
A lot or greater difficulty walking, using equipment/assistance	16.7 (11.7 – 21.7)	49.5 (41.9 – 57.0)	53.0 (44.7 – 61.3)	47.3 (37.2-57.5)	73.8 (62.9-82.4)	-
**Does not use mobility equipment or assistance**	**90.3 (88.6 – 92.1)**	**97.5 (97.1 – 97.9)**	**95.7 (94.7 – 96.6)**	**97.8 (97.3-98.2)**	**98.0 (97.5-98.4)**	-
Some or greater difficulty walking	33.8 (30.9 – 36.8)	7.5 (6.6 – 8.4)	26.4 (23.3 – 29.6)	8.5 (7.6-9.4)	16.9 (15.7-18.1)	-
A lot or greater difficulty walking	1.8 (0.9 – 2.6)	1.6 (1.3 – 2.0)	2.6 (1.6 – 3.7)	1.0 (0.7-1.4)	2.5 (2.0-3.0)	-
**Uses hearing aid‡**	**0.3 (0.0 – 0.9)**	**0.4 (0.2 – 0.5)**	**0.1 (0.0 – 0.3)**	**0.3 (0.1-0.5)**	**0.5 (0.3-0.8)**	**0.6 (0.3-1.1)**

‡Insufficient data for further analyses
